# Autoimmune Phenomena and New‐Onset Type 1 Diabetes Following SARS‐CoV‐2 Vaccination: A Personal Perspective

**DOI:** 10.1002/iid3.70419

**Published:** 2026-04-27

**Authors:** Kimihiko Sugaya

**Affiliations:** ^1^ Department of Molecular Imaging and Theranostics Institute for Quantum Medical Science, National Institutes for Quantum Science and Technology (QST) Chiba Japan

## Abstract

A molecular biologist whose wife suddenly developed type I diabetes in the summer of 2022 addresses the possible risk of developing autoimmune disease from messenger RNA (mRNA) vaccination against SARS‐CoV‐2. This essay also provides a record of the tough fight against autoimmune disease continuing to the present day, the prospect for therapeutics to control type I diabetes, and the author's for standing by his wife.

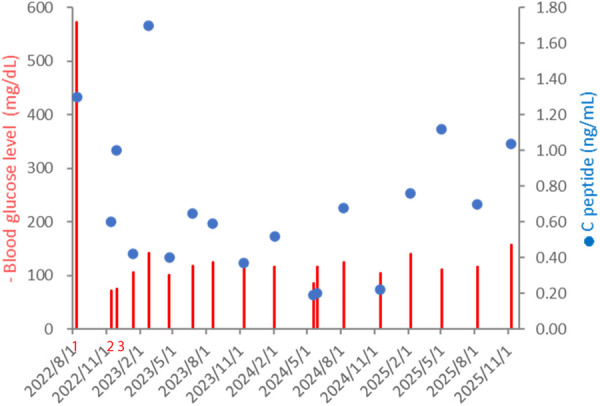

To the Editor,

A molecular biologist whose wife suddenly developed type I diabetes (T1D) in the summer of 2022 addresses the possible risk of developing autoimmune disease from messenger RNA (mRNA) vaccination against SARS‐CoV‐2. This essay also provides a record of the tough fight against autoimmune disease continuing to the present day, the prospect for therapeutics to control T1D, and the author's behavior for standing by his wife.

## 1 Onset

**Figure 1:**
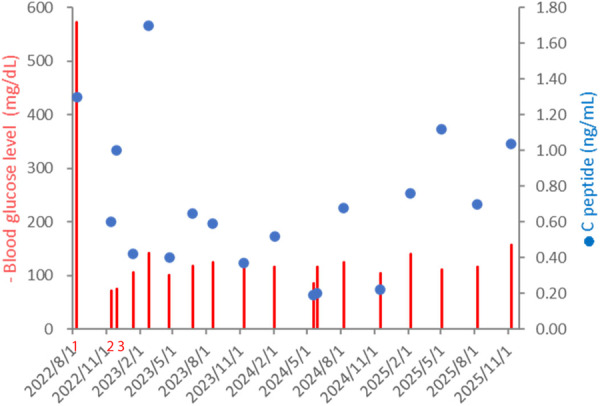
Changes in the blood glucose and C‐peptide levels of the author's wife. The red 1 indicates the day (August 12, 2022) the author's wife was diagnosed as having type I diabetes and the injections of insulin also began. The red 2 represents the start date (November 13, 2022) of the application and use of a FreeStyle Libre (Abbott) continuous glucose monitoring device. Finally, the red 3 indicates the start date (December 23, 2022) of the therapeutic immunosuppressant baricitinib (Olumiant), a JAK inhibitor, administered for her alopecia areata.

The author's 58‐year‐old wife suddenly developed T1D in the summer of 2022. Initially, she thought she had summer fatigue, but after a medical examination at a clinic, findings of a serum glucose level of nearly 600 mg/dL and positivity for the autoantibody against glutamic acid decarboxylase resulted in immediate detailed examination, diagnosis of T1D, and initiation of insulin injections (Figure 1). In T1D, insulin production by pancreatic β cells is faulty, and although the mechanistic details of β‐cell destruction in T1D remain unclear, T1D is considered an autoimmune disease. T1D is progressive, and ultimately, insulin is not produced due to depletion of β cells. When looking back at that time with some regret, I realize that my wife was thirsty and appeared to be in the early stage of diabetic ketoacidosis.

## 2 COVID‑19 Vaccine and Autoimmunity

Both of us were vaccinated against SARS‐CoV‐2 at the same time with the same kind of vaccine. Table 1 represents our personal vaccination record against SARS‐CoV‐2 and my wife's chronological development of the autoimmune diseases that occurred after vaccination. While the mRNA vaccine against SARS‐CoV‐2 brought great benefit to people worldwide, my wife suddenly developed alopecia and atopic dermatitis in succession after her second vaccination. Because both dermatoses appeared to be autoimmune diseases, I suspected vaccination against SARS‐CoV‐2 as the culprit. We decided to change vaccine manufacturer from Moderna to Pfizer, but just after the third vaccination, she developed yet another dermatosis, leukoderma vulgaris (white spots on face). Finally, T1D appeared in the summer of 2022. Thereafter, I found reports of new‐onset T1D being noticed after COVID‐19 vaccination [1, 2], and a review summarized new‐onset autoimmune phenomena occurring after COVID‐19 vaccination [3]. A genome‐wide association study has already revealed eight regions related to the pathogenesis of alopecia, and surprisingly, six genome regions of those were involved in other autoimmune diseases including T1D [4]. In 2024, a cohort study in Korea found no increased risk for most autoimmune connective tissue diseases after mRNA vaccination [5]. However, it also pointed out that booster doses were associated with increased risk of alopecia areata, psoriasis, and rheumatoid arthritis (RA). A recent study investigated whether two doses of the mRNA COVID‐19 vaccine influenced blood cytokine levels associated with major T helper cell populations and emphasized the safety of the mRNA COVID‐19 vaccine [6]. However, the authors of this article also recommended the requirement of large‐scale follow‐up studies to validate the immunological effects of COVID‐19 vaccination on the onset of autoimmune diseases in a broader population. As such, an association between mRNA vaccination and the onset of autoimmune diseases appears to be very complicated. Along with carefully considering the timing and number of vaccine doses, and any history of autoimmune diseases, a global epidemiologic investigation is still needed to better clarify this relation. The risk of developing autoimmune diseases, including T1D, appears to be increased following COVID‐19 infection [7]. It is also important to acknowledge that many individuals may have been exposed to the virus and undergone subtle immune changes without apparent infection or symptoms, and that new‐onset autoimmune diseases following vaccination may result from multifactorial mechanisms.

**Table 1:** COVID‐19 vaccinations and disease state of the author's wife.

COVID‐19 vaccination	Date	Disease state
1st, mRNA (Moderna)	July 9, 2021	
2nd, mRNA (Moderna)	August 6, 2021	
	September 2021	Alopecia
	September 2021	Atopic dermatitis
3rd, mRNA (Pfizer)	March 8, 2022	
	April 2022	Leukoderma vulgaris
	July 2022	Type 1 diabetes
4th, recombinant protein (Novavax)	November 8, 2022	
5th, recombinant protein (Novavax)	October 6, 2023	

## 3 From Fear to Relief

Honestly, just after my wife developed T1D, I was afraid of the further onset of autoimmune disease such as connective tissue disease including RA. Use of an immunosuppressive agent to help treat T1D was not allowed in Japan at that time and remains so even now. However, good news came from the field of dermatology. Baricitinib (Olumiant), an inhibitor of the Janus kinase family of enzymes (JAKs) with selectivity for JAK1 and JAK2, is currently in clinical development for the treatment of RA and other inflammatory disorders [8]. In Japan, baricitinib was first authorized as an oral therapeutic drug for RA in 2017 and then for atopic dermatitis in December 2020, SARS‐CoV‐2 pneumonia in April 2021, and alopecia areata in June 2022. We were lucky to be able to use baricitinib just after this authorization. Now, in November 2025, my wife's alopecia has almost completely recovered. Insulin is produced by proteolytic processing of the precursor proinsulin. The ability to secrete insulin is estimated by measuring C‐peptide produced by this proteolytic process. Figure 1 depicts the changes in my wife's C‐peptide levels after she developed T1D. Fortunately and surprisingly, her pancreatic β cells still appear to be producing insulin at 3 years after T1D onset.

## 4 Moving Forward

Though the effect of baricitinib is not clear, among the immunosuppressants administered to patients with T1D, anti‐CD3 monoclonal antibody teplizumab can delay the development of T1D. In 2022, the US FDA approved intravenous injection of teplizumab to delay T1D progression in people who already produce diabetes‐related autoantibodies and in people older than 8 years old with problems controlling blood sugar [9]. Another JAK inhibitor, ruxolitinib, also showed an effect in a T1D patient with a pathogenic mutation in signal transducer and activator of transcription 1 (*STAT1*), which confirms the diagnosis of STAT1 gain‐of‐function disease and allowed him to stop insulin injections [10].

I hope my wife's pancreatic β cells remain functional for as long as possible. I truly expect that elucidation of the detailed mechanisms underlying β‐cell loss in T1D patients will result in the development of novel medical treatments to prevent β‐cell destruction. Chimeric antigen receptor T‐cell therapy, which utilizes genetically engineered T cells to treat cancer, also appears to be an attractive therapy for autoimmune disease, though its appearance in clinical practice may take more time [11, 12].

For our fourth vaccination against SARS‐CoV‐2, we choose Novavax's recombinant protein vaccine instead of an mRNA type (Table 1), even though I have experienced no side effects after any of my vaccinations. I keenly hope that as many choices of vaccine type as possible can be provided. At least against SARS‐CoV‐2, Novavax's vaccine does not appear to be inferior to the mRNA‐type vaccines [13].

## Author Contributions

**Kimihiko Sugaya:** conceptualization, project administration, supervision, visualization, writing – original draft, writing – review and editing.

## Funding

The author has nothing to report.

## Consent

The author obtained the consent of his wife to present the data shown in the manuscript.

## Conflicts of Interest

The author declares no conflicts of interest.

## Data Availability

The author has nothing to report.
